# Exploring research training needs of oral healthcare professionals at Prince Sattam Bin Abdulaziz University, Saudi Arabia: a cross-sectional study

**DOI:** 10.3389/fdmed.2026.1645170

**Published:** 2026-02-11

**Authors:** Nasser Raqe Alqhtani, Ali Robaian, Abdullah Saad Alqahtani, Abdullah Alshehri, Abdullah Ali Alqahtani, Shahad Saleh Alghannam, Osamah F. Alqasem, Tarek Ahmed Soliman

**Affiliations:** 1 Department of Oral and Maxillofacial Surgery and Diagnostic Sciences, College of Dentistry, Prince Sattam Bin Abdulaziz University, Al-Kharj, Saudi Arabia; 2 Conservative Dental Sciences Department, College of Dentistry, Prince Sattam Bin Abdulaziz University, Al-Kharj, Saudi Arabia; 3 Department of Preventive Dentistry, College of Dentistry, Prince Sattam Bin Abdulaziz University, Al-Kharj, Saudi Arabia; 4 Dental Hospital, College of Dentistry, Prince Sattam Bin Abdulaziz University, Al-Kharj, Saudi Arabia

**Keywords:** needs assessment, research, dentistry, professional competence, development plan, Saudi Arabia

## Abstract

**Introduction:**

In alignment with Saudi Vision 2030, dental colleges are increasingly required to enhance research productivity and international academic standing. This study aimed to identify research training priorities among oral healthcare professionals at Prince Sattam Bin Abdulaziz University, Saudi Arabia.

**Methods:**

A validated questionnaire was distributed to faculty members and comprised four sections: demographics, prior research training, self-rated performance vs. perceived importance of research competencies, and preferred training delivery methods. Descriptive statistics were used to summarize responses.

**Results:**

A total of 45 out of 61 faculty members completed the survey (response rate: 73.8%). Participants’ evaluations of prior research training ranged from poor or fair to excellent, with the majority reporting training levels between good and very good. Research training priorities varied by academic rank, with assistant professors identifying a broader range of high-priority areas compared to associate professors and full professors.

**Conclusions:**

The findings support the development of targeted faculty development programs aligned with rank-specific research training priorities to strengthen research competencies and enhance institutional research capacity.

## 1 Introduction

Saudi Arabia is experiencing a transformative shift throughout all sectors of the nation. Education and healthcare are essential elements in the 2030 development plan. In alignment with Saudi Vision 2030, dental colleges are increasingly required to enhance their rankings by boosting research publications in high-impact international journals. Additionally, Saudi Vision 2030 emphasized how important research is to enhancing health systems (1–3). The research competencies for oral healthcare professionals at PSAU are ready for and in need of modernization. This modernization process cannot be done in seclusion but rather in collaboration between institutional goals and their need, to provide appropriately trained oral healthcare personnel able to compete in the international labor market.

The enhancement of research competencies among oral healthcare professionals involves a series of activities implemented by institutions to improve their skills in educational research. These activities can be a powerful tool in initiating and setting the direction for the modernization of institutional research plans (4–6). It fosters a favorable institutional environment and may involve fundamental research skills programs for junior staff as well as advanced programs for senior staff (7). A needs assessment is a planned way of gathering and analyzing data to identify the discrepancies between the current state and the desired state. Assessing the needs of oral healthcare professionals is a crucial endeavor of any university that believes in the value of people. Exploring researchers’ training needs guides decision-makers in addressing these requirements, thereby improving the research skills of participants and promoting a more conducive research environment (8–10).

Research experience is essential for evidence-based practice, as it develops skills in literature searching, data collection and analysis, and critical appraisal of evidence (11). Early training in research competencies has been linked to sustained professional academic engagement (12). Moreover, the utilization of international academic rankings has emerged as a prevalent and valuable method for comparing universities both domestically and globally (13). In 2012, a study in a public University in Malaysia reported that implementing a policy to motivate academic staff to enhance publishing output was successful, resulting in an increase in publications and an improvement in rankings from 2006 until February 14, 2011. Additionally, a review study analyzes research needs to identify key insights into the discipline. It has been reported that engaging stakeholders is advisable when carrying out surveys for learning needs assessment to prevent misinterpretations and ensure accurate conclusions (14).

The Times Higher Education ranking includes research quality and research environment as criteria for evaluating universities (15). Prince Sattam Bin Abdulaziz University (PSAU) is one of the modern universities in the Kingdom of Saudi Arabia. it was established in 2009. The vision statement of the university is “A university that is distinguished in education, competitive in research in support of knowledge economy, and effective in partnerships and social responsibility” to fulfill Saudi Vision 2030 (1, 16). Despite its newest foundation, its institutional ranking is currently 601–800th according to Times Higher Education 2025 (17).

The College of Dentistry at PSAU conducted a needs assessment to analyze the status and needs of oral healthcare professionals to improve their research competencies since research has become a priority of every higher education institution. Needs can improve performance or fix a certain shortcoming. It is a fact that improving faculty research skills cannot be done overnight, thus careful planning and prioritization of training must be efficiently considered. Specific topics in research must be identified clearly and rated according to their degree of need and urgency. There are a scarce number of studies regarding the assessment of research training needs in Saudi Arabia. This study intended to bridge gaps that hinder Oral healthcare Professionals from acquiring knowledge and skills based on their needs to contribute appropriately for improving ranking in the global university league. This study aimed to identify the key research competencies deemed essential by oral healthcare professionals, focusing on their knowledge and prioritization to enhance research productivity at the College of Dentistry- (PSAU), Saudi Arabia.

## 2 Methodology

### 2.1 Study design and ethical approval

This study utilized a cross-sectional survey design. A team of educational specialists in dentistry developed an online needs assessment tool to evaluate faculty members’ perceptions of the current research training program and identify key areas for enhancement. The study received ethical approval from the Standing Committee of Bioethics Research at PSAU (Approval No. SCBR-348/2024). All study procedures followed the Helsinki Declaration. Incomplete responses were excluded from analyses. This study followed the CHERRIES electronic survey guideline (18).

### 2.2 Study settings

The needs assessment questionnaire was created in accordance with survey methodology principles, emphasizing questionnaire length, existing literature on faculty development needs, and institutional requirements. All items were developed utilizing a structured, closed-ended format to facilitate standardized quantitative analysis. The questionnaire's content and face validity were evaluated independently by three experts in medical education. Their feedback led to improvements, which was then finalized. The content validity ratio and face validity index obtained were 0.83 and 0.92, respectively. The questionnaire was developed and administered in English, which is the official academic language at the institution; therefore, no translation was required. Minor contextual adaptations were made to align the items with the local academic structure and research environment. The questionnaire was tested with 30 participants to assess the clarity of the questions and to measure the time required for completion (7 min).

### 2.3 Participants and recruitment

The questionnaire was distributed to all full-time faculty members, through their official email addresses using a secure SurveyMonkey Professional link. Participants received a pre-notification and informed consent statement one week before the survey distribution and provided consent for the inclusion of their responses in aggregated analyses upon completion.

### 2.4 Questionnaire

The questionnaire was developed to identify priority research training needs for institutional planning purposes and was not intended as a psychometric measurement scale. The questionnaire comprised 27 items and required a maximum of 7 min for completion. The questionnaire was conducted from October to November 2024. The introductory section of the questionnaire requested responses from participants concerning their demographic characteristics, including gender, academic rank, department specialty, and number of published articles (5 questions). The demographic questions were followed by three main sections. The first part (2 questions) assessed participants’ opinions on research competency training and participation frequency. The second part of the questionnaire asked participants about their ’self-rated performance’ vs. ‘perceived importance’ on nineteen research competencies, including research study design (5 questions), conducting research (5 questions), research writing (6 questions), and research environment and management (3 questions). Self-rated performance was assessed using five Likert scales: 1 = little 2,  = average 3,  = good 4,  = approaching mastery 5,  = mastery/could teach others. Three Likert scales were employed to assess perceived importance of competencies 1: = Not at all important 2,  = Moderately important 3,  = Extremely important. The third section (1 question) evaluated the preferred approach for implementing the training activities.

### 2.5 Statistical analyses

Data were analyzed utilizing SPSS Statistics software version 27. Descriptive analyses were performed to summarize the characteristics of respondents and the distributions of their responses. In the needs-gap assessment, self-performance ratings were divided into lower levels (scores 1–3) and higher levels (scores 4–5), whereas perceived priority ratings were classified as lower (scores 1–2) or higher (score 3). The Chi-Square test was used to analyze and identify the academic rank differences in self-performance and their priority.

## 3 Results

### 3.1 Characteristics of participants

Out of 88 faculty members, 52 completed the survey. The teaching assistant (*n* = 7) was excluded from the study due to its low sample size relative to the total number of teaching assistants (*n* = 27) in the College. Accordingly, Out of 61 college faculty, 45 faculty (professors, associate professors, and assistant professors) completed the survey, resulting in a response rate of 73.7% (3 out of 5 professors, 15 out of 20 associate professors, and 27 out of 36 assistant professors). The majority were assistant professors (60%), followed by associate professors (33.3%), and professors (6.7%). Gender-wise distribution revealed (68.8%) male and (31.2%) female responders. 28.9% of the faculty published more than twenty articles, 48.9% of the faculty published 11–20 articles, and 22.2% published 1–10 articles. The assistant professor (46.7%) participated in 6–10 articles, the associate professor (77.8%) participated in more than 16 articles, and the professor (75%) participated more than 20 articles (Table 1).

**Table 1:** Characteristics of faculty who participated in this study (*n* = 45).

Variables		Frequency	Percentages (%)
Gender	Male	31	68.8
	Female	14	31.2
Academic position	Professor	3	6.7
	Associate professor	15	33.3
	Assistant professor	27	60
Number of published articles	More than 20	13	28.9
	16–20	4	8.9
	11–15	18	40
	6–10	8	17.8
	1–5	2	4.4
	Never	0	0
Attended training sessions number in research competencies in the last five years	Frequently (8 or more per year)	2	4.4
	Often (5–7 per year)	3	6.6
	Sometimes (3–4 per year)	20	44.4
	Rarely (1–2 per year)	17	37.8
	Never	3	6.8

### 3.2 Faculty’s rating of the current research training activities

Participants rated their previous training on research competencies as poor or fair (11.54% to 28.85%), good (42.31% to 51.32%), very good (9.62% to 21.5%), and excellent (1.92–3.85%). The participants’ responses across five levels of participation (never, rarely, sometimes, often, and frequently) were 6.8%, 37.8%, 44.4%, 6.6%, and 4.4%, respectively (Table 1). 66.7% of professors and 46.7% of associate professors participate in four or more workshops annually. Among assistant professors, 57.1% reported attending training workshops infrequently or not at all. No statistically significant differences (*P* > 0.5) were observed between participants’ satisfaction and their level of participation in the training programs.

### 3.3 Research study design competencies

Table 2 showed a statistically significant difference between self-performance and priority for S2, S3, and S5. The faculty needs improvements regarding S3 and S5. Faculty self-performance of S2 is statistically significantly higher than the priority. Items in the Knowledge and Priority columns with percentages exceeding 40% were classified as high need and high priority (Table 3). Upon examining the data, the professors and associate professors recognized one high-priority competency (S5), while assistant professors identified four competencies (S2, S3, S4, and S5) as high-priority, based on knowledge percentages and priority levels exceeding 40%.

**Table 2:** Participants’ self-performance and perceived importance (percentages) categorized by academic positions (research study design).

Competencies	Rating	Academic positions (percentages)			Total percentages
		Professor	Associate. professor	Assistant professor	
S1. Conducting Literature Searches effectively	LK	1/3 (33.3%)	3/15 (20%)	10/27 (37.1%)	14/45 (31.1%)
	HP	0/3 (0%)	9/15 (60%)	13/24 (54.2%)	22/42 (52.3%)
	*P* value	0.190	0.042*	0.234	0.043*
S2. Review a particular area of research and write a comprehensive literature review	LK	0/3 (0%)	4/15 (26.6%)	16/27 (59.3%)	20/45 (44.4%)
	HP	1/3 (33.3%)	8/15 (53.3%)	12/24 (50%)	21/42 (50%)
	*P* value	0.196	0.034*	0.183	0.083
S3. Formulate a clear research question or testable hypothesis	LK	1/3 (33.3)	4/15 (26.7%)	15/27 (55.6%)	20/45 (44.4%)
	HP	0/3 (0%)	9/15 (60%)	16/24 (66.7%)	25/42 (59.5%)
	*P* value	0.196	0.024*	0.074	0.058
S4. Choose a research study design for the research question	LK	1/3 (33.3%)	5/15 (33.3%)	18/27 (66.7%)	24/45 (53.3%)
	HP	1/3 (33.3)	10/15 (66.7%)	18/24 (75%)	29/42 (69.1%)
	*P* value	0.981	0.024*	0.095	0.061
S5. Design and implement the best sampling strategy for your study	LK	3/3 (100%)	7/15 (46.7%)	20/27 (74.1%)	30/45 (66.7%)
	HP	2/3 (66.7%)	10/15 (66.7%)	17/24 (70.8%)	29/42 (69.1%)
	*P* value	0.023*	0.041*	0.091	0.094

LK, lower knowledge; HP, higher priority.

*p value is statistically significant.

**Table 3:** Participants’ self-performance and perceived importance (percentages) categorized by academic positions (conducting research study).

Competencies	Rating	Academic positions (Percentages)			Total percentages
		Professor	Associate. professor	Assistant professor	
C1. Design and implement the best measurement approach for your study	LK	1/3 (33.3%)	7/15 (46.7%)	19/27 (70.4%)	27/45 (60%)
	HP	0/3 (0%)	6/15 (40%)	17/24 (70.8%)	24/42 (54.7%)
	*P* value	0.864	0.671	0.973	0.097
C2. Knowledge of ethical clinical practice guidelines.	LK	1/3 (33.33)	4/15 (26.6. %)	10/27 (37.1%)	15/45 (33.3%)
	HP	0/3 (0%)	9/15 (60%)	16/24 (66.7%)	25/42 (59.5%)
	*P* value	0.861	0.043*	0.050*	0.042*
C3. Data records and management	LK	3/3 (100%)	9/15 (60%)	18/27 (66.7%)	30/45 (66.7%)
	HP	0/3 (0%)	5/15 (33.3%)	9/24 (37.5%)	14/42 (33.3%)
	*P* value	˂0.001*	0.032*	˂0.001*	0.034*
C4. Design and implement the best data analysis for your study	LK	2/3 (66.7%)	8/15 (53.3%)	21/27 (77.8%)	31/45 (68.9%)
	HP	3/3 (100%)	9/15 (60%)	14/24 (58.3%)	26/42 (61.9%)
	*P* value	0.078	0.341	0.059	0.239
C5. Interpreting analyzed data	LK	2/3 (66.7%)	8/15 (53.3%)	19/27 (70.4%)	29/45 (64.4%)
	HP	3/3 (100%)	11/15 (73.3%)	15/24 (62.5%)	29/42 (69.1%)
	*P* value	0.082	0.043*	0.085	0.192

LK, lower knowledge; HP, higher priority.

*p value is statistically significant.

### 3.4 Conducting research study competencies

Table 3 showed a statistically significant difference between self-performance and priority for C1, C4, and C5. The faculty needs improvements regarding all these items. The professors recognized two high-priority competencies (C4 and C5), while associate and assistant professors identified three competencies (C1, C4, and C5) as high-priority, based on knowledge below 40% and priority exceeding 40%.

### 3.5 Research writing competencies

Table 4 showed a statistically significant difference between self-performance and priority for W5 and W6. The faculty needs improvements regarding all these items. The professors identified two high-priority competencies (W2, and W6), associate professors identified three competencies (W2, W5, and W6), and assistant professors identified three competencies (W1, W3, and C5) as high-priority competencies.

**Table 4:** Participants’ self-performance and perceived importance (percentages) categorized by academic positions (research writing skills).

Competencies	Rating	Academic positions (Percentages)			Total percentages
		Professor	Associate professor	Assistant professor	
W1. Drafting a manuscript	LK	0/3 (0%)	4/15 (26.7%)	12/27 (44.4%)	16/45 (35.6%)
	HP	1/3 (33.3%)	7/15 (46.7%)	16/24 (66.6%)	24/42 (57.1%)
	*P* value	0.832	0.045*	0.05*	0.05*
W2. Grant Proposal Writing in Discipline Research	LK	2/3 (66.67%)	6/15 (40%)	10/27 (37.1%)	18/45 (40%)
	HP	2/3 (66.7%)	7/15 (46.7%)	15/24 (62.5%)	24/42 (57.1)
	*P* value	1	0.092	0.023*	0.042*
W3.Addressing journals’ reviewer comments	LK	0/3 (0%)	4/15 (26.7%)	18/27 (66.7%)	22/45 (48.8%)
	HP	1/3 (33.3%)	10/15 (66.7%)	16/24 (66.7%)	28/42 (64.2%)
	*P* value	0.832	˂ 0.001*	1	0.086
W4.Choosing the appropriate journal	LK	0/3 (0%)	4/15 (26.7%)	8/27 (29.6%)	12/45 (26.7%)
	HP	0/3 (0%)	5/15 (33.3%)	15/24 (62.5%)	20/42 (47.6%)
	*P* value	1	0.124	˂ 0.001*	0.050*
W5.International Publications	LK	0/3 (0%)	6/15 (40%)	20/27 (74.1%)	26/45 (57.8%)
	HP	3/3 (100%)	10/15 (66.7%)	16/24 (66.7%)	27/42 (64.3%)
	*P* value	˂ 0.001*	0.021*	0.129	0.175
W6. References resources	LK	3/3 (100%)	7/15 (46.7%)	19/27 (70.4%)	29/45 (64.4%)
	HP	3/3 (100%)	6/15 (40%)	13/24 (54.2%)	22/42 (52.4%)
	*P* value	1	0.239	0.067	0.168

LK, lower knowledge; HP, higher priority.

*p value is statistically significant.

### 3.6 Research environment and time management competencies

Table 5 showed a statistically significant difference between self-performance and priority for M2. Associate and assistant professors rated M2 competency as a high-priority competency based on lower knowledge and priority percentages greater than 40%.

**Table 5:** Participants’ self-performance and perceived importance (percentages) categorized by academic positions (research environment and management).

Competencies	Rating	Academic positions (Percentages)			Total percentages
		Professor	Associate. professor	Assistant professor	
M1. Prioritizing research tasks	LK	1/3 (33.3%)	5/15 (33.3%)	10/27 (37.1%)	16/45 (35.6%)
	HP	0/3 (0%)	8/15 (53.3%)	11/24 (45.8%)	20/42 (45.2%)
	*P* value	0.193	0.231	0.196	0.199
M2. Research time management	LK	3/3 (100%)	9/15 (60%)	23/27 (85.2%)	35/45 (77.8)
	HP	1/3 (33.3%)	8/15 (53.3%)	14/24 (58.3%)	23/42 (54.8%)
	*P* value	˂ 0.001*	0.751	˂ 0.001*	0.043*
M3.Contribute effectively to the research team	LK	1/3 (33.3%)	5/15 (33.3%)	8/27 (29.6%)	14/45 (31.1%)
	HP	1/3 (33.3%)	9/15 (60%)	10/24 (41.6%)	20/42 (47.6%)
	*P* value	0.998	0.05*	0.092	0.083

*p value is statistically significant.

### 3.7 Top high-priority competencies categorized by academic ranks

Professors identified five competencies as high-priority competencies: one in research study design competencies (S5), two in conducting research study competencies (C4, and C5), and two in research writing competencies (W2, and W6). The associate professor identified eight competencies as high-priority competencies: one in research study design competencies (S5), three in conducting research study competencies (C1, C4, and C5), three in research writing competencies (W2, W5, and W6), and one in research environment and time management competencies (M2). The assistant professors identified eleven competencies as high-priority competencies: four in research study design competencies (S2, S3, S4, and S5), three in conducting research study competencies (C1, C4, and C5), three in research writing competencies (W1, W3, and W5), and one in research environment and time management competencies (M2) (Table 6).

**Table 6:** Top high-priority competencies categorized by academic ranks.

Competencies	Academic positions		
	Full professor	Associate professor	Assistant professor
S1. Conducting Literature Searches effectively			
S2. Review a particular area of research and write a comprehensive literature review			❖
S3. Formulate a clear research question or testable hypothesis			❖
S4. Choose a research study design for the research question			❖
S5. Design and implement the best sampling strategy for your study	✓	▪	❖
C1. Design and implement the best measurement approach for your study		▪	❖
C2. Knowledge of ethical clinical practice guidelines.			
C3. Data records and management			
C4. Design and implement the best data analysis for your study	✓	▪	❖
C5. Interpreting analyzed data	✓	▪	❖
W1. Drafting a manuscript			❖
W2. Grant Proposal Writing in Discipline Research	✓	▪	
W3.Addressing journals’ reviewer comments			❖
W4.Choosing the appropriate journal			
W5.International Publications		▪	❖
W6. References resources	✓	▪	
M1. Prioritizing research tasks			
M2. Research time management		▪	❖
M3.Contribute effectively to the research team			

### 3.8 Participants’ preferences for training activities delivery

Online webinars (70.83%) and face-to-face interactive group sessions (62.50%) are the preferred highest methods for delivery of the training sessions.

## 4 Discussion

The College of Dentistry recognizes the significance of research in knowledge, innovation, and social advancement. Research training needs must be assessed to ensure faculty have the necessary research competencies (19–22). Needs assessment aids in analysis and prioritization of faculty development to improve education quality (23). Sometimes faculty members perceiving their knowledge as limited and prioritizing these items accordingly. Conversely, at other times, faculty may perceive their knowledge to be significant and their priorities regarding these items to be correspondingly high (23, 24). A previous study (25) indicated that self-assessment among health professions educators frequently reveals gaps between perceived competence and perceived importance, thereby emphasizing areas that require focused professional development. This study aimed to identify the key research competencies deemed essential by oral healthcare professionals, focusing on their knowledge and prioritization to enhance research productivity at the College of Dentistry- (PSAU), Saudi Arabia. Consequently, any item within the Knowledge and Priority areas of this study that received a percentage exceeding 40% was classified as a high priority (23, 24).

Prince Sattam University organizes a variety of training programs including research skills on a yearly schedule distributed before the academic year begins. Up to 50% rated the training program as acceptable and 11.54%–28.85% rated it as poor to fair. These findings necessitate a modernization process. The justification is due to the unsuitable timing of the training activities and the non-mandatory nature of these activities.

The study findings’ indicated that assistant professor demonstrated a high-priority research training needs compared to professors and associate professors. This finding reflects differences in self-reported competency levels and perceived priorities across academic ranks. Additionally, the results showed that eight research competencies were rated as higher priority, indicating areas where additional training and professional development may be beneficial. These findings emphasize the importance of aligning faculty development initiatives with identified training needs to strengthen research capacity at the institutional level. These training priorities may be considered within the context of ongoing developments in healthcare systems and evolving academic promotion requirements at the institutional level. Our findings align with prior research indicating that junior faculty demonstrate increased motivation to engage in structured professional development as they strive to establish their scholarly identities (26). Furthermore, previous studies indicate that early-career faculty priorities pedagogical training more than senior faculty, who generally rely on their accumulated experience instead of formal training (27, 28). Additionally, partnership with the national Saudi Commission for Healthcare Specialties to build a postgraduate training program, “Saudi Board for Family Dental Medicine,” would improve health practitioners’ research skills. Also, the College of Dentistry is preparing to switch from a traditional to a competency-based approach, and new curricula emphasizing faculty development. Since research and scholarship are essential for oral healthcare professionals, these findings support the value of implementing tailored research training activities that are aligned with academic rank. Most participants choose face-to-face or online training programs. The findings support previous evidence suggesting academics prefer interactive professional development. This shows that academic personnel prefer interactive training that incorporates the application and practice of acquired knowledge (29, 30).

By addressing research training needs, it would improve educational research environment, produce relevant and impactful research outcomes, contribute to the broader societal development goals and consequently boosting the institution's academic standing. The study findings provide institution policymakers customized research plans for each academic rank depending on their knowledge and priority level to execute effective training programs. Clear research plans characterize a “vital and sustainable” research environment. Furthermore, it is essential to continue conducting research on training needs assessments to support sustainable global progress, particularly in relation to SDG 4 (14). This study has limitations; the findings are based on self-reported data, which may not fully reflect actual research competencies since participants may overestimate or underestimate their abilities or respond in accordance with perceived expectations. Although the study included a significant percentage of eligible faculty members; however, the overall sample size and the limited number of subgroups restrict the statistical power of comparisons between ranks. Therefore, subgroup findings must be interpreted cautiously and may not be generalizable beyond the institutional context. The questionnaire was developed for institutional needs assessment and evaluated for face and content validity; however, comprehensive psychometric validation, including internal consistency or factor analysis, was not conducted. Future research should integrate qualitative methodologies, involve teaching assistant personnel, and enhance the validation of the measurement instrument to obtain a deeper understanding of faculty training requirements.

## 5 Conclusion

This study provides tailored dental research training programs. They highlighted key areas for research training competencies that should be prioritized to strengthen the research capabilities of faculty members, resulting in better research outcomes. The findings indicate differences in perceived training priorities among academic ranks, with assistant professors recognizing a broad range of development areas, whereas associate professors and professors emphasized more focused, rank-specific competencies. These insights may support the strategic planning of faculty development activities designed to enhance institutional research capacity.

## Data Availability

The original contributions presented in the study are included in the article/Supplementary Material, further inquiries can be directed to the corresponding author.
